# Disparity in Lung Cancer Screening Among Smokers and Nonsmokers in China: Prospective Cohort Study

**DOI:** 10.2196/43586

**Published:** 2023-03-14

**Authors:** Le Wang, Youqing Wang, Fei Wang, Yumeng Gao, Zhimei Fang, Weiwei Gong, Huizhang Li, Chen Zhu, Yaoyao Chen, Lei Shi, Lingbin Du, Ni Li

**Affiliations:** 1 Department of Cancer Prevention Zhejiang Cancer Hospital, Institute of Basic Medicine and Cancer (IBMC), Chinese Academy of Sciences Hangzhou China; 2 Zhejiang Key Laboratory of Diagnosis & Treatment Technology on Thoracic Oncology (Lung and Esophagus) Hangzhou China; 3 Office of Cancer Screening National Cancer Center/National Clinical Research Center for Cancer/Cancer Hospital, Chinese Academy of Medical Sciences and Peking Union Medical College Beijing China; 4 Chinese Academy of Medical Sciences Key Laboratory for National Cancer Big Data Analysis and Implement, Chinese Academy of Medical Sciences and Peking Union Medical College Beijing China; 5 Hwa Mei Hospital, University of Chinese Academy of Sciences Ningbo China; 6 Kecheng District People's Hospital of Quzhou Quzhou China; 7 Zhejiang Provincial Centre for Disease Control and Prevention Hangzhou China; 8 Department of Radiology Zhejiang Cancer Hospital, Institute of Basic Medicine and Cancer (IBMC), Chinese Academy of Sciences Hangzhou China

**Keywords:** lung cancer, screening, smoker, nonsmoker

## Abstract

**Background:**

Low-dose computed tomography (LDCT) screening is effective in reducing lung cancer mortality in smokers; however, the evidence in nonsmokers is scarce.

**Objective:**

This study aimed to evaluate the participant rate and effectiveness of one-off LDCT screening for lung cancer among smokers and nonsmokers.

**Methods:**

A population-based prospective cohort study was performed to enroll participants aged between 40 and 74 years from 2013 to 2019 from 4 cities in Zhejiang Province, China. Participants who were evaluated as having a high risk of lung cancer from an established risk score model were recommended to undergo LDCT screening. Follow-up outcomes were retrieved on June 30, 2020. The uptake rate of LDCT screening for evaluated high-risk participants and the detection rate of early-stage lung cancer (stage 0-I) were calculated. The lung cancer incidence, lung cancer mortality, and all-cause mortality were compared between the screened and nonscreened groups.

**Results:**

At baseline, 62.56% (18,818/30,079) of smokers and 6% (5483/91,455) of nonsmokers were identified as high risk (*P*<.001), of whom 41.9% (7885/18,818) and 66.31% (3636/5483) underwent LDCT screening (*P*<.001), respectively. After a median follow-up of 5.1 years, 1100 lung cancer cases and 456 all-cause death cases (116 lung cancer death cases) were traced. The proportion of early-stage lung cancer among smokers was 60.3% (173/287), which was lower than the proportion of 80.3% (476/593) among nonsmokers (*P*<.001). Among smokers, a higher proportion was found in the screened group (72/106, 67.9%) than the nonscreened group (56/114, 49.1%; *P*=.005), whereas no significance was found (42/44, 96% vs 10/12, 83%; *P*=.20) among nonsmokers. Compared with participants who were not screened, LDCT screening in smokers significantly increased lung cancer incidence (hazard ratio [HR] 1.39, 95% CI 1.09-1.76; *P*=.007) but reduced lung cancer mortality (HR 0.52, 95% CI 0.28-0.96; *P*=.04) and all-cause mortality (HR 0.47, 95% CI 0.32-0.69; *P*<.001). Among nonsmokers, no significant results were found for lung cancer incidence (*P*=.06), all-cause mortality (*P*=.89), and lung cancer mortality (*P*=.17).

**Conclusions:**

LDCT screening effectively reduces lung cancer and all-cause mortality among high-risk smokers. Further efforts to define high-risk populations and explore adequate lung cancer screening modalities for nonsmokers are needed.

## Introduction

### Background

Lung cancer is the second most commonly diagnosed cancer and the leading cause of cancer-related death worldwide; in 2020, there were an estimated 2.2 million new cases and 1.8 million deaths [1]. In 2019, more than one-third of all newly diagnosed lung cancers and approximately 40% of global cancer-related deaths occurred in China [1], where lung cancer was the leading cause of disability-adjusted life years and years of life lost [2], resulting in a high disease and socioeconomic burden. These trends underscore the need for continued efforts to improve lung cancer outcomes.

Screening with low-dose computed tomography (LDCT) is effective in reducing lung cancer mortality in rigorously randomized controlled trials such as the National Lung Screening Trial (NLST) and the Dutch-Belgian lung cancer screening trial (Nederlands Leuvens Screening Onderzoek [NELSON]); however, such trials have high LDCT uptake rates exceeding 90% [3,4]. These high uptake rates are not attainable in real-world settings; thus, evidence from real-world screening settings with imperfect uptake rates is crucial to inform appropriate and effective screening policies. Moreover, current findings are only applicable to smokers, who are considered as a high-risk lung cancer subgroup. The prevalence of nonsmoking-related lung cancer in East Asian countries is higher than that in Europe and the United States [5]. In China, tobacco smoking is responsible for 75% of lung cancer cases in men and 18% in women [6]. Therefore, the effectiveness of LDCT screening among nonsmokers at high risk of lung cancer still needs to be evaluated.

### Objectives

The Cancer Screening Program in Urban China (a large public health service project) conducted in 2012 included both smokers and nonsmokers and targeted 5 types of cancer (lung cancer, female breast cancer, esophageal and gastric cancer, colorectal cancer, and liver cancer) [7]. In Zhejiang, the smoking rate of people aged ≥15 years was over 20%, which is relatively low among all provinces in China [8]. Nevertheless, Zhejiang Province has a higher lung cancer incidence than the national average [9,10]. These phenomena make Zhejiang an appropriate setting to study the effectiveness of lung cancer screening among smokers and nonsmokers. Using lung cancer screening data from the Cancer Screening Program in Urban China conducted in the first 7 years between October 2013 and September 2019 in Zhejiang, we evaluated the effectiveness of one-off LDCT screening in the early detection and reduction of lung cancer mortality and all-cause mortality among smokers and nonsmokers, respectively.

## Methods

### Study Design and Participants

We performed a population-based prospective study using the framework of the Cancer Screening Program in Urban China [11]. Trained staff called or visited residents aged 40-74 years living in selected communities of the participating cities. Patients with a history of cancer and those undergoing treatment for other serious medical and surgical diseases were excluded. For this screening program, only participants at high risk of lung cancer (refer to Risk Assessment Procedure) were recommended to undergo LDCT free of charge at a tertiary-level hospital designated by the program. All participants provided written informed consent.

**Figure 1:**
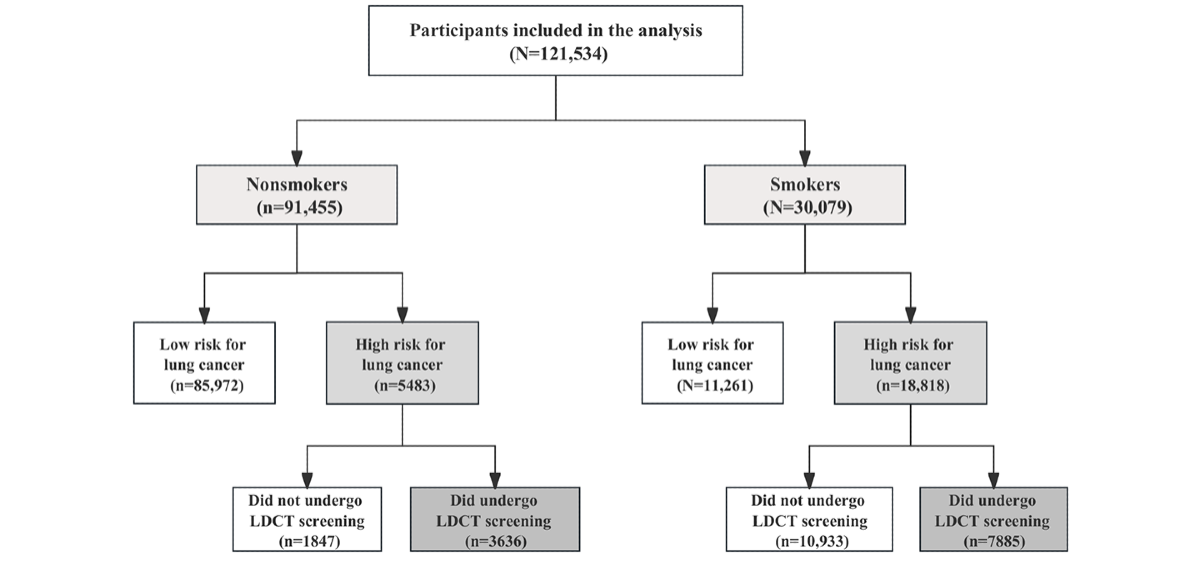
Flow diagram of the study population. LDCT: low-dose computed tomography.

We used data from lung cancer screening conducted between October 2013 and September 2019 in Zhejiang Province, which covered 4 cities (Hangzhou, Ningbo, Quzhou, and Jinhua). Overall, 121,534 eligible individuals participated in the lung cancer screening program (Figure 1). For this study, smokers were defined as those who had previously smoked or were currently smoking tobacco more than once per day for at least 6 months. The smokers and nonsmokers enrolled in this study were classified into 3 groups: the screened group (high-risk participants who underwent LDCT screening), the nonscreened group (high-risk participants who did not undergo LDCT screening), and the low-risk group.

### Ethics Approval

The study was approved by the ethics committee of the Chinese Academy of Medical Sciences’s Cancer Hospital (approval number 15-070/997) and Zhejiang Cancer Hospital (approval number IRB-2022-271).

### Risk Assessment Procedure

Eligible participants completed a cancer-related risk assessment questionnaire designed by the Cancer Screening Program in Urban China before LDCT that included questions regarding cigarette smoking history, occupational exposure to hazardous substances, frequent exercise, chronic respiratory diseases, family history of lung cancer, dietary intake of fresh vegetables in the previous year, and passive smoking. We adopted the sex-specific risk score systems derived from the Harvard Cancer Risk Index to evaluate the risk of lung cancer [12,13]. Each risk factor was assigned a score by an expert panel based on the magnitude of its association with lung cancer. Cumulative risk scores were calculated and divided by the average risk score in the general population, yielding final individual relative risks. Individuals with a relative risk >2.0 or aged ≥50 years with a smoking index of ≥400 (number of cigarettes smoked per day multiplied by years of smoking) were defined as being at high risk for lung cancer [12,13].

Individuals labeled as being at high risk for lung cancer were recommended to undergo a free LDCT scan with ≥16 slices at a tertiary-level hospital designated by the program. These participants then underwent a process of shared decision-making that included information about the potential benefits and harms of screening with LDCT to ensure that their decisions on LDCT scans were based on their free will.

### Follow-up Data

To account for potential immortal time bias, where individuals in the screened group had to survive (be alive and event free) until the LDCT scan was conducted, the cohort entry date was defined as the date of screening in the screened group. For individuals in the nonscreened group, the cohort entry date was estimated based on the screening date of the individual in the screened group whose risk assessment date was closest to that of the nonscreened group. Time to lung cancer occurrence was calculated from the cohort entry date until the earliest occurrence of lung cancer, death, or administrative censoring (June 30, 2020). Accordingly, time to lung cancer death or all-cause death was calculated from the cohort entry date until death or administrative censoring, whichever occurred first.

### Outcome Assessment and Quality Control

The primary outcomes of interest were incidence of lung cancer, lung cancer mortality, and all-cause mortality. The secondary outcomes were the proportion of early-stage lung cancer (stage 0-I) and the participation rate of LDCT. Lung cancer was defined according to the International Classification of Diseases (10th revision) and was coded as C34. Outcome data were retrieved from national linkages, including the cancer registry system and death surveillance system, every 6 months.

Paper-based standardized documentation forms (epidemiological questionnaire and LDCT report) were collected from trained staff and physicians. Form validity was checked and entered into the data management system by trained study staff. A consistency check was performed, and if inconsistencies were identified, errors were corrected by retrieving the original records. Each participant had a unique identification number that was used to track all individual-related documentation forms. All data were transmitted to the Central Data Management Team of the National Cancer Center of China, where databases were constructed and analyzed.

### Definitions of Covariates

Covariates from the baseline survey included demographic characteristics (age: 40-54 years and 55-74 years; sex; education level: low [primary school or below], medium [primary school to high school], and high [high school or above]; and BMI), lifestyle factors (smoking status, passive smoking, occupational exposure to hazardous substances, and frequent exercise), family history of lung cancer, and baseline comorbidities (chronic respiratory diseases, digestive diseases, hepatobiliary diseases, hypertension, diabetes, and hyperlipidemia). Passive smoking referred to involuntary inhalation of tobacco smoke. Occupational exposure to hazardous substances included occupational exposure to asbestos, rubber, dust, pesticides, radiation, beryllium, uranium, and radon for at least 1 year. Frequent exercises were defined as exercises conducted at least 3 times per week for ≥30 minutes each. Respiratory diseases included pulmonary tuberculosis, chronic bronchitis, emphysema, asthmatic bronchiectasis, silicosis, and pneumoconiosis.

### Statistical Analysis

Baseline study population characteristics were summarized using frequencies and percentages for categorical variables and arithmetic means and SDs for continuous variables. Baseline factors were compared between the nonsmoker and smoker groups using a 2-tailed Student *t* test or chi-square test. Group-specific participation rates of LDCT screening for high-risk populations by common factors were also calculated. Multivariable logistic regression was used to explore the potential determinants of the participant rate of LDCT screening. For lung cancer outcomes, cumulative incidence was estimated using the cumulative incidence function, accounting for the competing risk of mortality. Lung cancer mortality was estimated using the cumulative mortality function, accounting for the competing risk of death from other causes. Gray tests were used to assess differences between the groups for cumulative lung cancer incidence and lung cancer mortality. All-cause mortality was calculated using Kaplan-Meier analysis with a log-rank test to assess differences between groups. Owing to the small number of deaths among nonsmokers, we only used a Cox proportional hazards model among smokers to calculate the hazard ratios (HRs) and 95% CI of LDCT screening with lung cancer incidence, mortality, and all-cause mortality. All hypothesis tests were 2 sided. Statistical analyses were performed using R (version 3.5.1; R Foundation for Statistical Computing) and SAS (version 9.4; SAS Institute Inc), and *P*<.05 was considered statistically significant.

## Results

### Baseline Characteristics

In total, 121,534 participants aged 40-74 years who were enrolled in the program were included in this study; of them, 75.3% (n=91,453) were nonsmokers and 24.7% (n=30,079) were smokers. Compared with nonsmokers, smokers were older; were more likely to be male; were more likely to have medium and high educational levels; were more overweight and obese; and had more occupational exposure to hazardous substances, passive smoking, family history of lung cancer, chronic respiratory diseases, digestive diseases, hepatobiliary diseases, hypertension, hyperlipidemia, and diabetes but were less likely to exercise frequently (*P*<.001; Table 1). Comparisons of baseline characteristics by risk assessment and screening status for smokers and nonsmokers are, respectively, presented in Multimedia Appendices 1 and 2.

**Table 1:** Baseline characteristics of the study population (N=121,534).

Characteristics				Overall^a^ (N=121,534)		Nonsmoker group^b^ (n=91,455)		Smoker group^c^ (n=30,079)		*P* value^d^	
**Demographic characteristics**											
	**Age (years), mean (SD)**		56.5 (8.3)		56.4 (8.4)		56.8 (8.0)		<.001		
		40-54, n (%)	49,948 (41.10)		38,166 (41.73)		11,782 (39.17)				
		55-74, n (%)	71,586 (58.90)		53,289 (58.27)		18,297 (60.83)				
	**Sex, n (%)**										<.001
		Male	51,623 (42.48)		22,499 (24.60)		29,124 (96.83)				
		Female	69,911 (57.52)		68,956 (75.40)		955 (3.17)				
	**Education, n (%)**										<.001
		Low	36,691 (30.19)		28,925 (31.63)		7766 (25.82)				
		Medium	70,489 (58.00)		51,977 (56.83)		18,512 (61.54)				
		High	14,354 (11.81)		10,553 (11.54)		3801 (12.64)				
	**BMI (kg/m^2^), n (%)**										<.001
		<18.5	3294 (2.71)		2645 (2.90)		649 (2.16)				
		18. 5-24	68,948 (56.82)		52,997 (58.05)		15,951 (53.12)				
		24-28	40,842 (33.66)		29,630 (32.45)		11,212 (37.33)				
		≥28	8250 (6.80)		6031 (6.61)		2219 (7.39)				
**Lifestyle factors**											
	**Occupational exposure to hazardous substances, n (%)**										<.001
		No	106,361 (87.52)		82,044 (89.71)		24,317 (80.84)				
		Yes	15,173 (12.48)		9411 (10.29)		5762 (19.16)				
	**Passive smoking, n (%)**										<.001
		No	74,087 (61.11)		63,153 (69.15)		10,934 (36.55)				
		Yes	47,158 (38.89)		28,173 (30.85)		18,985 (63.45)				
	**Frequent exercise, n (%)**										<.001
		No	64,019 (52.68)		45,355 (49.59)		18,664 (62.05)				
		Yes	57,515 (47.32)		46,100 (50.41)		11,415 (37.95)				
	**Family history of lung cancer, n (%)**										<.001
		No	98,370 (88.69)		74,851 (90.17)		23,519 (84.30)				
		Yes	12,539 (11.31)		8159 (9.83)		4380 (15.70)				
**Baseline comorbidity**											
	**Chronic respiratory diseases, n (%)**										<.001
		No	105,151 (86.52)		81,371 (88.97)		23,780 (79.06)				
		Yes	16,383 (13.48)		10,084 (11.03)		6299 (20.94)				
	**Digestive diseases, n (%)**										<.001
		No	90,832 (74.74)		70,115 (76.71)		20,717 (68.88)				
		Yes	30,702 (25.26)		21,340 (23.29)		9362 (31.12)				
	**Hepatobiliary diseases, n (%)**										<.001
		No	90,549 (74.51)		70,159 (76.71)		20,390 (67.79)				
		Yes	30,985 (25.49)		21,296 (23.29)		9689 (32.21)				
	**Hypertension, n (%)**										<.001
		No	78,772 (71.20)		60,271 (72.72)		18,501 (66.66)				
		Yes	31,858 (28.80)		22,605 (27.28)		9253 (33.34)				
	**Hyperlipidemia, n (%)**										<.001
		No	91,603 (82.81)		69,381 (83.72)		22,222 (80.07)				
		Yes	19,018 (17.19)		13,487 (16.28)		5531 (19.93)				
	**Diabetes, n (%)**										<.001
		No	101,246 (91.52)		76,417 (92.21)		24,829 (89.46)				
		Yes	9379 (8.48)		6455 (7.79)		2924 (10.54)				

^a^Overall: 200 participants without information on BMI, 289 participants without information on passive smoking, 10,625 participants without information on family history of lung cancer, 10,904 participants without information on hypertension, 10,913 participants without information on hyperlipidemia, and 10,909 participants without information on diabetes.

^b^Nonsmokers: 152 participants without information on BMI, 129 participants without information on passive smoking, 8445 participants without information on family history of lung cancer, 8579 participants without information on hypertension, 8587 participants without information on hyperlipidemia, and 8583 participants without information on diabetes.

^c^Smokers: 48 participants without information on BMI, 160 participants without information on passive smoking, 2180 participants without information on family history of lung cancer, 2325 participants without information on hypertension, 2326 participants without information on hyperlipidemia, and 2326 participants without information on diabetes.

^d^*P* values were generated by using the chi-square or *t* test (2-tailed) by smoking status.

### Participation Rates for LDCT Screening for High-risk Participants

Among smokers, 62.56% (18,818/30,079) were identified as being at high risk for lung cancer, which was significantly higher than 5.8% (5483/94,455) among nonsmokers (*P*<.001). Among the high-risk participants, 41.9% (7885/18,818) underwent LDCT screening among smokers, which was significantly lower than 66.31% (3636/5483) among nonsmokers (*P*<.001; Figure 1). The data on LDCT participation by subgroup are shown in Table 2. Results from multivariable logistic regression models showed that female participants (odds ratio [OR] 1.33, 95% CI 1.11-1.58; *P*<.001), older participants (OR 1.24, 95% CI 1.16-1.33; *P*<.001), participants with occupational exposure to hazardous substances (OR 1.50, 95% CI 1.38-1.62; *P*<.001), participants with a family history of lung cancer (OR 1.74, 95% CI 1.60-1.90; *P*<.001), participants with chronic respiratory diseases (OR 1.40, 95% CI 1.29-1.51; *P*<.001), participants with digestive diseases (OR 1.24, 95% CI 1.16-1.33; *P*<.001), participants with hepatobiliary diseases (OR 1.35, 95% CI 1.25-1.45; *P*<.001), participants with hyperlipidemia (OR 1.19, 95% CI 1.10-1.30; *P*<.001), and participants with diabetes (OR 1.16, 95% CI 1.04-1.29; *P*<.001) had higher LDCT participation rates among smokers. Among nonsmokers, participants with occupational exposure to hazardous substances (OR 1.37, 95% CI 1.19-1.58; *P*<.001), participants with a history of passive smoking (OR 1.22, 95% CI 1.04-1.44; *P*<.001), participants with a family history of lung cancer (OR 1.83, 95% CI 1.62-2.08; *P*<.001), and participants with hepatobiliary diseases (OR 1.37, 95% CI 1.21-1.56; *P*<.001) had higher LDCT participation rates.

**Table 2:** Participation rates of low-dose computed tomography (LDCT) screening for high-risk population by smoking status (n =24,301).

Characteristics			Nonsmoker group^a^					Smoker group^b^				
			Participants at high risk, n	Underwent LDCT, n (%)	*P* value^c^	OR^d^ (95% CI)	Participants at high risk, n		Underwent LDCT, n (%)	*P* value^c^		OR^d^ (95% CI)
**Demographic characteristics**												
	**Age (years)**				.005						<.001	
		40-54	2374	1623 (68.37)		—^e^	7217		2779 (38.51)			Reference
		55-74	3109	2013 (64.75)		—	11,601		5106 (44.01)			1.24 (1.16-1.33)
	**Sex^f^**				—						<.001	
		Male	0	0 (0)		—	18,178		7524 (41.39)			Reference
		Female	5483	3636 (66.31)		—	640		361 (56.41)			1.33 (1.11-1.58)
	**Education**				.65						.48	
		Low	1889	1261 (66.75)		—	5304		2247 (42.36)			—
		Medium	3054	2026 (66.34)		—	11,540		4833 (41.88)			—
		High	540	349 (64.63)		—	1974		805 (40.78)			—
	**BMI**				.42						<.001	
		<18.5	187	114 (60.96)		—	445		174 (39.10)			—
		18.5-24	3156	2107 (66.76)		—	10,066		4089 (40.62)			—
		24-28	1736	1153 (66.42)		—	6882		2978 (43.27)			—
		≥28	393	257 (65.39)		—	1393		634 (45.51)			—
**Lifestyle factors**												
	**Occupational exposure to hazardous substances**				<.001						<.001	
		No	3716	2338 (62.92)		Reference	14,712		5668 (38.53)			Reference
		Yes	1767	1298 (73.46)		1.37 (1.19-1.58)	4106		2217 (53.99)			1.50 (1.38-1.62)
	**Passive smoking**				.001					<.001		
		No	949	586 (61.75)		Reference	5735		2140 (37.31)			—
		Yes	4463	2998 (67.17)		1.22 (1.04-1.44)	12,935		5649 (43.67)			—
	**Frequent exercise**				.76						.41	
		No	4205	2784 (66.21)		—	13,486		5676 (42.09)			—
		Yes	1278	852 (66.67)		—	5332		2209 (41.43)			—
	**Family history of lung cancer**				<.001						<.001	
		No	2098	1238 (59.01)		Reference	13,583		5213 (38.38)			Reference
		Yes	3200	2305 (72.03)		1.83 (1.62-2.08)	3764		2255 (59.91)			1.74 (1.60-1.90)
**Baseline comorbidity**												
	**Chronic respiratory diseases**				.12						<.001	
		No	333	234 (70.27)		—	13,440		4875 (36.27)			Reference
		Yes	5150	3402 (66.06)		—	5378		3010 (55.97)			1.40 (1.29-1.51)
	**Digestive diseases**				.03						<.001	
		No	2158	1394 (64.60)		—	12,246		4519 (36.90)			Reference
		Yes	3325	2242 (67.40)		—	6572		3366 (51.22)			1.24 (1.16-1.33)
	**Hepatobiliary diseases**				<.001						<.001	
		No	2233	1355 (60.7)		Reference	12,186		4361 (35.79)			Reference
		Yes	3250	2281 (70.2)		1.37 (1.21-1.56)	6632		3524 (53.14)			1.35 (1.25-1.45)
	**Hypertension**				.80						<.001	
		No	3254	2162 (66.44)		—	11,319		4630 (40.90)			—
		Yes	1506	995 (66.07)		—	5736		2612 (45.54)			—
	**Hyperlipidemia**				—						<.001	
		No	3174	2068 (65.15)		—	13,458		5367 (39.88)			Reference
		Yes	1583	1086 (68.60)		—	3596		1873 (52.09)			1.19 (1.10-1.30)
	**Diabetes**				.21						<.001	
		No	4301	2864 (66.59)		—	15,225		6356 (41.75)			Reference
		Yes	457	291 (63.68)		—	1829		885 (48.39)			1.16 (1.04-1.29)

^a^Nonsmokers: 11 participants without information on BMI, 71 participants without information on passive smoking, 185 participants without information on family history of lung cancer, 723 participants without information on hypertension, 726 participants without information on hyperlipidemia, and 725 participants without information on diabetes.

^b^Smokers: 32 participants without information on BMI, 148 participants without information on passive smoking, 1471 participants without information on family history of lung cancer, 1763 participants without information on hypertension, 1764 participants without information on hyperlipidemia, and 1764 participants without information on diabetes.

^c^*P* values were generated by using the chi-square and Cochran-Armitage test statistics for differences where appropriate; missing values are not included.

^d^OR: odds ratio. ORs are presented for variables with significance in the multivariate logistic regression.

^e^The variable is excluded from the final model and OR is not available.

^f^All nonsmokers were female .

### Overall Lung Cancer Incidence and Mortality

After a median follow-up time of 5.1 years (IQR 3.1-5.9 years), 377 lung cancer cases, 202 all-cause death cases, and 67 lung cancer death cases were observed among smokers, and 733 lung cancer cases, 254 all-cause death cases, and 49 lung cancer death cases were observed among nonsmokers (Table 3). The crude lung cancer incidence density was 277.57 (95% CI 250.92-307.05) per 100,000 person-years in smokers and 178.51 (95% CI 166.05-191.92) per 100,000 person-years in nonsmokers (Table 4), resulting in a crude rate ratio of 1.555 (95% CI 1.547-1.563). The crude all-cause mortality rate and lung cancer mortality rate in smokers was 147.89 (95% CI 128.83-169.76) per 100,000 person-years and 49.05 (95% CI 38.61-62.33) per 100,000 person-years, respectively, which were higher than 61.63 (95% CI 54.50-69.69) per 100,000 person-years and 11.89 (95% CI 8.99-15.73) per 100,000 person-years in nonsmokers, yielding a crude rate ratio of 2.4 (95% CI 2.382-2.417) and 4.126 (95% CI 4.057-4.195), respectively. Among lung cancer patients with available data on stage and histological information, the proportion of stage 0-I was 60.3% (173/287) among smokers and lower than the proportion of 80.3% (476/593) among nonsmokers (*P*<.001), and the proportion of adenocarcinoma was 61.2% (194/317) among smokers and lower than the proportion of 90.2% (607/673) among nonsmokers (*P*<.001; Table 3). Subgroup analyses showed that the proportion of stage 0-I was 67.9% (72/106) in the screened group, which was significantly higher than 49.1% (56/114) in the nonscreened group (*P*=.005) among smokers, and no significance was found of the proportion of stage 0-Ibetween the screened group (42/44, 96%) and the nonscreened group (10/12, 83%) among nonsmokers (*P*=.20).

**Table 3:** Lung cancer incidence cases and deaths among all participants (N=121,534).

			Nonsmoker^a^					Smoker^b^			
			All (n=91,455)	Low risk (n=85,972)	Nonscreened (n=1847)	Screened (n=3636)	All (n=30,379)		Low risk (n=11,261)	Nonscreened (n=10,933)	Screened (n=7885)
**Incident cases, n**											
	All cases		733	662	17	54	377		86	148	143
	**Stage** **, n (%)**										
		0-I	476 (80.3)	424 (79)	10 (83.3)	42 (95.5)	173 (60.3)		45 (67.2)	56 (49.1)	72 (67.9)
		II	24 (4.1)	23 (4.2)	1 (8.3)	0 (0)	19 (6.6)		3 (4.5)	8 (7)	8 (7)
		III	32 (5.4)	31 (5.8)	1 (8.3)	0 (0)	47 (16.4)		8 (11.9)	22 (19.3)	17 (15)
		IV	61 (10.3)	59 (11)	0 (0)	2 (4.5)	48 (16.7)		11 (16.4)	28 (24.6)	9 (8.5)
	**Histological type, n (%)**										
		Adenocarcinoma	607 (90.2)	546 (89.1)	16 (94.1)	45 (93.8)	194 (61.2)		54 (71.1)	59 (48.4)	81 (68.1)
		Squamous cell carcinoma	34 (5.1)	33 (5.4)	0 (0)	1 (2.1)	79 (24.9)		16 (21.1)	39 (32)	24 (20.2)
		Small-cell carcinoma	13 (1.9)	13 (2.1)	0 (0)	0 (0)	31 (9.8)		4 (5.3)	18 (14.8)	8 (7.6)
		Others	19 (2.8)	21 (3.4)	1 (5.9)	2 (4.2)	13 (4.1)		2 (2.6)	6 (4.9)	5 (4.2)
**Death cases, n**											
	All-cause death cases		254	246	3	5	202		64	102	36
	Lung cancer death cases		49	48	1	0	67		13	40	14

^a^Nonsmokers: 140 lung cancer cases without information on stage and 60 lung cancer cases without information on histological type.

^b^Smokers: 90 lung cancer cases without information on stage and 60 lung cancer cases without information on the histological type.

**Table 4:** Lung cancer incidence density and mortality rate by subgroups.

					Nonsmoker													Smoker									
					Cases, n		Crude rate^a^ (95% CI)			Adjusted rate^a,b^ (95% CI)			*P* value^c^				Cases, n				Crude rate^a^ (95% CI)			Adjusted rate^a,b^ (95% CI)		*P* value	
**Lung cancer incidence density**																											
	Overall			733		178.51 (166.05-191.92)			179.78 (166.14-194.54)			N/A^d^				377				277.57 (250.92-307.05)			219.86 (157.77-306.40)		N/A		
	**Subgroup**													<.001^c^													<.001^c^
		Low-risk group	662			170.23 (157.74-183.70)		173.28 (159.64-188.08)			N/A				86				157.80 (127.74-194.94)			144.60 (67.76-308.57)			N/A		
		Nonscreened group	17			223.72 (139.08-359.88)		129.32 (80.39-208.03)			.06^e^				148				306.10 (260.55-359.61)			132.65 (112.91-155.84)			<.001		
		Screened group	54			382.33 (292.82-499.21)		223.37 (171.03-291.72)			N/A				143				433.71 (368.14-510.96)			389.49 (249.18-608.81)			N/A		
**All-cause mortality rate**																											
	Overall			254		61.63 (54.50-69.69)			72.62 (63.54-83.01)			N/A				202				147.89 (128.84-169.76)			74.83 (54.98-103.73)		N/A		
	**Subgroup**													.29^c^													<.001^c^
		Low-risk group	246			63.04 (55.63-71.43)		73.09 (63.92-83.58)			N/A				64				117.10 (91.65-149.61)			84.83 (37.31-192.89)			N/A		
		Nonscreened group	3			39.29 (12.67-121.81)		22.56 (7.28-69.95)			.89^e^				102				210.0 (172.9-255.0)			91.03 (74.97-110.53)			<.001^e^		
		Screened group	5			35.02 (14.57-84.13)		20.74 (8.63-49.86)			N/A				36				107.93 (77.86-149.63)			46.03 (33.16-63.88)			N/A		
**Lung cancer mortality rate**																											
	Overall			49		11.89 (8.99-15.73)			15.98 (11.85-21.55)			N/A				67				49.05 (38.61-62.33)			32.4 (15.75-66.64)		N/A		
	**Subgroup**													.43^c^													<.001^c^
		Low-risk group	48			12.3 (9.3-16.3)		23.2 (18.6-29.1)			N/A				13				25.7 (16.0-41.3)			23.8 (13.8-41.0)			N/A		
		Nonscreened group	1			13.1 (1.8-93.0)		7.5 (1.1-53.4)			.17^e^				40				82.3 (60.4-112.3)			35.7 (26.2-48.6)			<.001^e^		
		Screened group	0^f^			0^f^		0^f^			N/A				14				41.97 (24.86-70.87)			17.7 (10.47-29.94)			N/A		

^a^Rate is the number of cases per 100,000 person-year.

^b^Rate is adjusted by age group and gender.

^c^*P* value is generated by comparing the 3 groups (low-risk group, nonscreened group, and screened group).

^d^N/A: not applicable.

^e^*P* value is generated by comparing the 2 groups (nonscreened group and screened group).

^f^No lung cancer death cases were reported for the group.

### Lung Cancer Incidence and Mortality in Smokers

**Figure 2:**
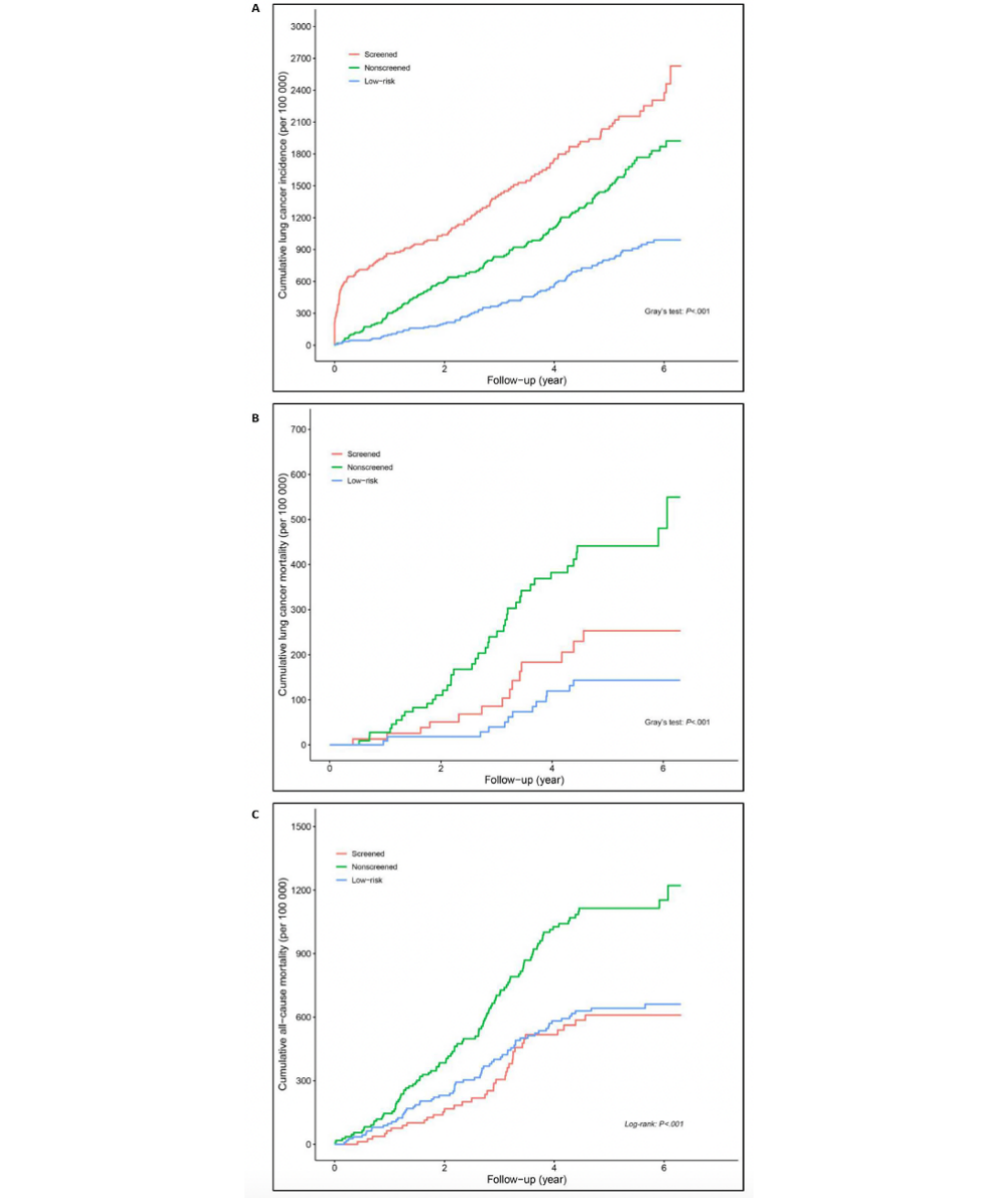
(A) Cumulative intensity of lung cancer incidence, (B) cumulative lung cancer mortality, and (C) cumulative all-cause mortality in smokers.

For smokers, the crude lung cancer incidence densities in the low-risk, nonscreened, and screened groups were 157.80 (95% CI 127.74-194.94) per 100,000 person-year, 306.10 (95% CI 260.55-359.61) per 100,000 person-year, and 433.71 (95% CI 368.14-510.96) per 100,000 person-year, respectively (*P*<.001; Figure 2A-C; Table 4). After adjusting for potential confounders, participants at high risk had significantly higher lung cancer incidence intensity (HR 2.20, 95% CI 1.72-2.82; *P*<.001), lung cancer mortality (HR 2.93, 95% CI 1.60-5.37; *P*<.001), and all-cause mortality rate (HR 1.42, 95% CI 1.06-1.91; *P*=.02) than participants at low risk, and participants in the screened group had significantly higher lung cancer incidence (HR 1.39, 95% CI 1.09-1.76; *P*=.007) but lower lung cancer mortality (HR 0.52, 95% CI 0.28-0.96; *P*=.04) and all-cause mortality (HR 0.47, 95% CI 0.32-0.69; *P*<.001) than the nonscreened group (Multimedia Appendix 3).

### Lung Cancer Incidence and Mortality in Nonsmokers

**Figure 3:**
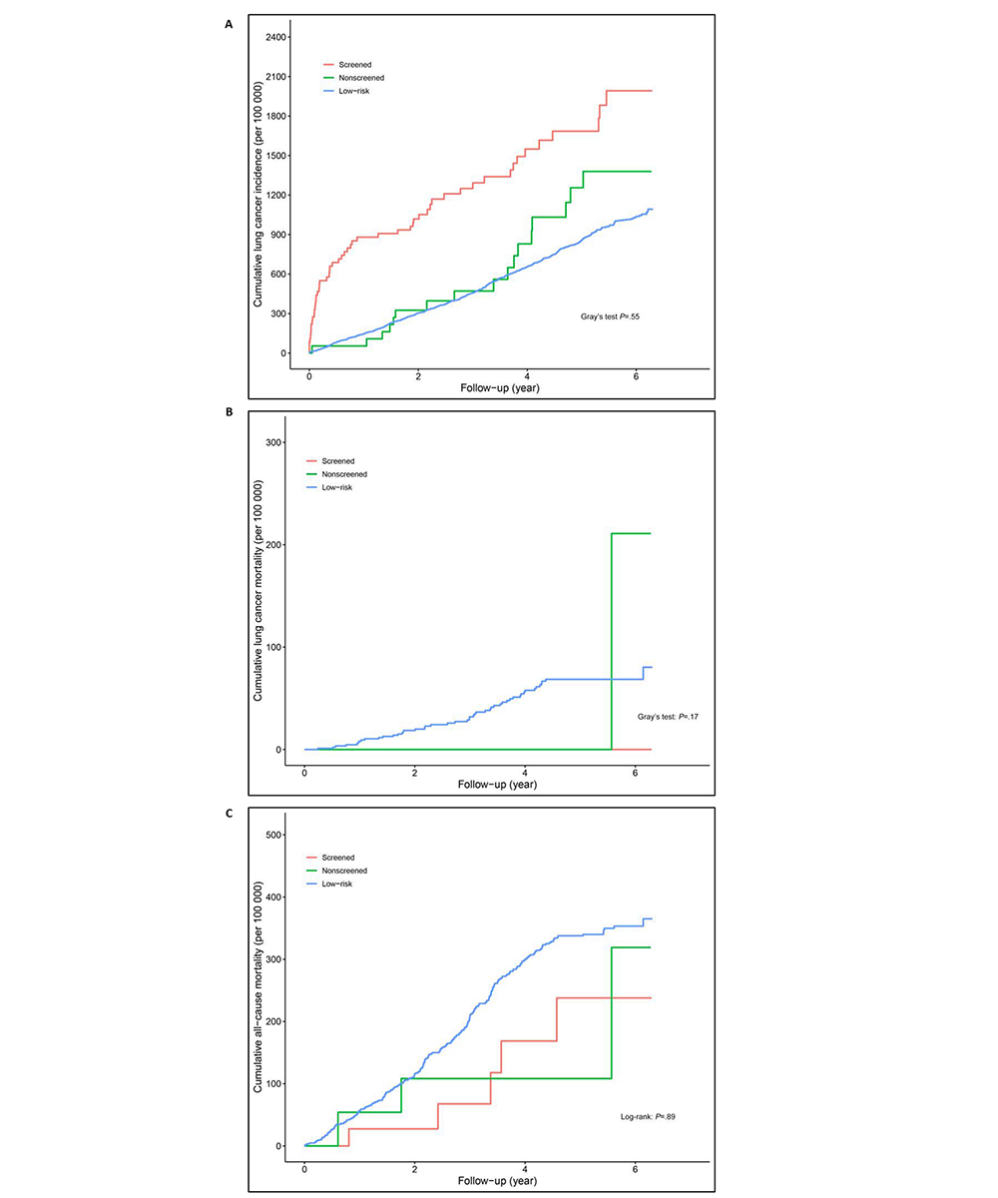
(A) Cumulative intensity of lung cancer incidence, (B) cumulative lung cancer mortality, and (C) cumulative all-cause mortality in nonsmokers.

For nonsmokers, lung cancer incidence densities in the low-risk, nonscreened, and screened groups were 170.23 (95% CI 157.74-183.70) per 100,000 person-year, 223.72 (95% CI 139.08-359.88) per 100,000 person-year, and 382.33 (95% CI 292.82-499.21) per 100,000 person-year, respectively. No significant differences were detected in lung cancer intensity (*P*=.06), all-cause mortality (*P*=.89), and lung cancer mortality (*P*=.17) between the screened and nonscreened groups (Table 4; Figure 3).

## Discussion

### Principal Findings

In this multicenter-based prospective cohort study involving ≥120,000 participants, we found a higher participation rate of LDCT screening among nonsmokers than among smokers (3636/5483, 66.31% vs 7885/18,818, 41.9%). Statistically significant reduced lung cancer mortality and all-cause mortality were observed among smokers in the screened group compared with those in the nonscreened group, with a higher proportion of early-stage lung cancer (72/106, 67.9% in the screened group and 56/114, 49.1% in the nonscreened group). However, we failed to find a significant mortality reduction among screened nonsmokers, relative to nonscreened nonsmokers.

### Comparison With Prior Work

The LDCT screening participation rate for nonsmokers was higher than that for smokers, as reported in other studies [13,14]. Studies have consistently shown that smokers have lower recognition of tobacco harm, lower health awareness, or preferred not to be notified of their status of lung cancer [14-18]. Women accounted for the majority of nonsmokers, and women had better awareness of cancer prevention than men [19]. This called for a multipronged approach to enhance engagement and extensively scale up lung cancer screening among smokers in China. As expected, nonsmokers and smokers with lung cancer risk factors (such as occupational exposure to hazardous substances, family history of lung cancer, and history of hepatobiliary diseases) had better LDCT screening compliance, as these disease-related factors may raise people’s awareness of lung cancer. For individuals with high lung cancer risk but low screening compliance, promotion of and training on the benefits and need for lung cancer screening are urgently required. Therefore, health education should target these residents to develop good living habits and improve cancer awareness.

As expected, a higher lung cancer incidence density was detected in the high-risk and screened groups, and most lung cancers among the nonsmoker participants were detected in the early stages (476/593, 80.3% were in stage 0-I), in contrast to those among smokers (173/287, 60.3% were in stage 0-I). Moreover, our study showed that the proportion of early-stage lung cancer was also high in the nonscreened group (10/12, 83% vs 56/114, 49.1%). A multicenter hospital-based study in China reported that the proportion of patients with stage I lung cancer was only 19% [20]. This disparity implies that screening programs involving health education, even without the provision of free LDCT examination, might increase the detection rate of early-stage lung cancer when it is still curable.

In our study, the most frequent histological type was adenocarcinoma, with a proportion of 90.2% (607/673) among nonsmokers and 61.2% (194/317) among smokers. East Asian female never-smokers tend to develop adenocarcinoma, with the majority developing from oncogenic mutations [21]. Significantly higher proportions of adenocarcinoma among nonsmokers can be explained by the higher proportion of women among nonsmokers. In addition, in the screened group, the proportion of stage 0-I disease among nonsmokers was higher than that among smokers (42/44, 96% vs 72/106, 67.9%). Similar results were found in a population screened in Korea (92.7% vs 63.6%) [22]. These findings strongly suggest that LDCT screening may be an effective strategy to detect early-stage lung cancer in nonsmokers and smokers in Asia, even though the incidence density of lung cancer is lower in nonsmokers. Despite the poor prognosis of lung cancer, patients with stage I disease have a 5-year survival rate of >75% [23,24]. Therefore, our results establish the potential value of lung cancer screening programs for smokers and nonsmokers.

Over a median follow-up time of 5.1 years, among smokers who were evaluated as high risk, we found a remarkable 48% lung cancer mortality reduction by screening with LDCT. LDCT screening has high potential benefit in decreasing lung cancer mortality worldwide, as shown in the NLST, NELSON, and the German Lung Cancer Screening Intervention Trial studies, which demonstrated that LDCT screening reduced lung cancer mortality by 20%, 24%, and 26%, respectively [3,4,25]. The reduction in our study was greater because we focused on the smoker subgroup. In the Multicentric Italian Lung Detection trial, a similar reduction (39%) was observed in lung cancer–related mortality following LDCT screening among smoking high-risk participants with ≥20 pack-years [26]. As tobacco smoking is a major risk factor for lung cancer [27], we conclude that participants with a history of smoking are likely to derive the greatest benefit from screening. Unlike in trials, the participants were randomly allocated into the LDCT screening group and control group, whereas in our study, the nonscreened group was determined from high-risk participants who declined to participate in further LDCT examination. This indicates that individuals in the nonscreened group (who are at high risk of lung cancer but did not undergo an LDCT scan) were more likely to have poor health awareness and less likely to seek medical assistance. This could be explained by the lowest proportion of early-stage lung cancer, highest all-cause mortality rate, and highest lung cancer mortality rate among the nonscreened group, followed by the low-risk and screened groups. Therefore, we strongly recommend LDCT screening for lung cancer in regions with high smoking rates [28].

The promising effects of the LDCT screening program observed in this study may be because of sufficient medical resources and health care infrastructure in Zhejiang. Zhejiang is a coastal province with a relatively high economy level in China, of which the gross domestic product per capita was US $14,600 in 2020, which was 1.40 times that of the national average and 1.45 times that of the global average. Due to its rapidly growing economy and a well-developed medical system, cancer screening has been well received and undertaken since the 1970s [28,29]. With screening practices in place for decades, Zhejiang has successfully established relatively mature screening networks in local or primary care and has developed multidisciplinary teams of professionals, resulting in a successful and effective LDCT screening program.

We observed a significant 53% reduction in all-cause mortality following LDCT screening among smokers, which was higher than that reported by the NLST (6.7%); however, several European randomized controlled trials, such as the detection and screening of early lung cancer with novel imaging technology and NELSON trials [4,30], found no significant reduction. Much larger sample sizes are needed to detect a significant reduction in all-cause mortality [31]; the sample size in our study was adequate. The consistent reduction in lung cancer mortality and all-cause mortality that we observed in our study is reassuring and supports the accuracy of cause of death assignment and suggests no important harms of screening [32]. In addition to early detection of lung cancer, LDCT screening provides an opportunity to detect cardiovascular disease, pulmonary disease, and extrapulmonary neoplasms [33-35], as a beneficial reduction in all-cause mortality may result from clinically significant incidental findings.

We did not observe a significant reduction in either lung cancer mortality or all-cause mortality by screening for nonsmokers. This may be because (1) a high proportion of early-stage lung cancers might lead to a favorable prognosis and a limited number of deaths; (2) considering the inadequate sample size as well as the limited follow-up time, no lung cancer deaths were traced in the follow-up period of this study among nonsmokers; or (3) we lacked accurate risk stratification strategies of nonsmokers for lung cancer screening. In East Asia, approximately one-third of lung cancers are unrelated to smoking [36,37]. Between 60% and 80% of women with lung cancer in Asia are nonsmokers compared with between 15% and 20% in the United States and Europe [5]. The current recommendation in the United States is that nonsmokers should not be screened; however, this recommendation is based on modeling in a predominantly White population, making the conclusion likely not applicable to Asian countries with a higher proportion of lung cancers in nonsmokers [38,39]. Moreover, in a population-based ecological cohort study among young women in Taiwan of China who rarely smoke, a significant upward trend in lung cancer incidence after LDCT screening but a stable trend in mortality was observed [40], which is suggestive of screening-related lung cancer overdiagnosis. Nevertheless, LDCT screening decreased the lung cancer mortality rate (HR 0.41) among nonsmokers in Hitachi City, Japan [41], suggesting that LDCT screening is effective in preventing lung cancer death among nonsmokers. In this study, the high adherence to LDCT screening and the high proportion of early-stage lung cancer may reflect a high level of conscientiousness among nonsmokers, indicating that the screening program involving health education has been advantageous for nonsmokers. However, it remains unclear whether LDCT screening can help nonsmokers in reducing mortality. Hence, longer follow-up data are urgently required to evaluate the effects of LDCT screening among nonsmokers. A risk prediction model that is applicable to Asian populations to identify nonsmokers with a high risk of lung cancer for LDCT screening must be established.

### Limitations

Our study had some limitations. First, this study may not be representative of the entire general population of China, but it can provide a scientific basis for other regions with similar socioeconomic status. Second, this was a real-world study rather than a randomized controlled trial, which might have led to residual confounders. We admit that a health volunteer effect existed in our study. Our program increased the health awareness of all participants involved in the program, including those in the screened, nonscreened, and low-risk groups. This can explain why the early-stage distributions were higher among all groups than in the general population. These findings suggest that screening programs involving health education may also increase the early detection of lung cancer and potentially decrease disease-specific mortality. Third, the median follow-up of 5.1 years in this study might be insufficient to trace cases and achieve consistent findings. Therefore, additional studies with extended follow-up times should be conducted to further evaluate the screening effects. Fourth, the smoker group was not categorized into heavy and light smokers because of the limited number of cases, and male participants without available information on smoking history were excluded. Passive smoking was only considered for women, which potentially induced gender disparity in smokers and nonsmokers. Finally, the outcome data presented here were obtained from the cancer registry and death surveillance systems, which might have resulted in a misclassification bias. Nevertheless, the cancer registry data in Zhejiang definitively met the requirements of the International Agency for Research on Cancer and the International Association of Cancer Registries, and the data were included in *Cancer Incidence in Five Continents* (*Volume VIII-XI*) consecutively [42-44]. Thus, the data quality obtained from Zhejiang Province was reliable.

### Conclusions

In conclusion, our findings suggest effective reduction of all-cause and lung cancer mortality among smokers following LDCT screening, whereas evidence of LDCT screening effectiveness for nonsmokers is still insufficient. It is important to not only target smokers but also identify nonsmokers at high risk of developing lung cancer to implement lung cancer screening programs and maximize screening benefits in China. Our study has significant health service implications, thus providing promising evidence to support the implementation of a national lung cancer screening program and to define optimal guidelines for lung cancer screening in China.

## Appendix

Multimedia Appendix 1 Baseline characteristics of the study population of nonsmokers.

Multimedia Appendix 2 Baseline characteristics of the study population of smokers.

Multimedia Appendix 3 Effects of risk evaluation and low-dose computed tomography screening on lung cancer incidence, lung cancer mortality, and all-cause mortality among smokers.

## Abbreviations

HR: hazard ratio
LDCT: low-dose computed tomography
NELSON: Nederlands Leuvens Screening Onderzoek
NLST: National Lung Screening Trial
OR: odds ratio
