# Supplementary material for: Disparity in Lung Cancer Screening Among Smokers and Nonsmokers in China: Prospective Cohort Study
Source: JMIR Public Health Surveill. 2023 Mar 14;9:e43586. doi: 10.2196/43586 (PMC10131892; doi:10.2196/43586)
Supplement: Multimedia Appendix 1 [file publichealth_v9i1e43586_app1.docx]

## Table S1. Baseline characteristics of the study population of non-smokers ^a, b^

| **Characteristics** | **High-risk** | | | | **Low-risk  (N=85 792)** | **Standardized difference ^d^** |
| --- | --- | --- | --- | --- | --- | --- |
|  | **Total** **(N =5483)** | **Non-screened**  **(N=1847)** | **Screened (N=3636)** | **Standardized difference ^d^** |  |  |
| **Demographic characteristics** |  |  |  |  |  |  |
| **Age (years)** |  |  |  |  |  |  |
| 40-54 | 2374 (43.3) | 751 (40.7) | 1623 (44.6) | -0.08 | 35792 (41.6) | -0.034 |
| 55-74 | 3109 (56.7) | 1096 (59.3) | 2013 (55.4) |  | 50180 (58.4) |  |
| **Sex** |  |  |  | 0 |  | -0.842 |
| Male ^c^ |  |  |  |  | 22499 (26.2) |  |
| Female | 5483 (100) | 1847 (100) | 3636 (100) |  | 63473 (73.8) |  |
| **Education** |  |  |  | 0.026 |  | 0.078 |
| Low | 1889 (34.5) | 628 (34) | 1261 (34.7) |  | 27036 (31.4) |  |
| Medium | 3054 (55.7) | 1028 (55.7) | 2026 (55.7) |  | 48923 (56.9) |  |
| High | 540 (9.8) | 191 (10.3) | 349 (9.6) |  | 10013 (11.6) |  |
| **Body mass index** |  |  |  | 0.047 |  | 0.042 |
| <18·5 | 187 (3.4) | 73 (4) | 114 (3.1) |  | 2458 (2.9) |  |
| 18·5-24 | 3156 (57.7) | 1049 (57) | 2107 (58) |  | 49841 (58.1) |  |
| 24-28 | 1736 (31.7) | 583 (31.7) | 1153 (31.8) |  | 27894 (32.5) |  |
| ≥28 | 393 (7.2) | 136 (7.4) | 257 (7.1) |  | 5638 (6.6) |  |
| **Lifestyle factors** |  |  |  |  |  |  |
| **Occupational exposure to hazardous substances** | | |  | 0.225 |  | 0.603 |
| No | 3716 (67.8) | 1378 (74.6) | 2338 (64.3) |  | 78328 (91.1) |  |
| Yes | 1767 (32.2) | 469 (25.4) | 1298 (35.7) |  | 7644 (8.9) |  |
| **Passive smoking** |  |  |  | 0.091 |  | 1.322 |
| No | 949 (17.5) | 363 (19.9) | 586 (16.4) |  | 62204 (72.4) |  |
| Yes | 4463 (82.5) | 1465 (80.1) | 2998 (83.6) |  | 23710 (27.6) |  |
| **Frequent exercise** |  |  |  | 0.009 |  | -0.623 |
| No | 4205 (76.7) | 1421 (76.9) | 2784 (76.6) |  | 41150 (47.9) |  |
| Yes | 1278 (23.3) | 426 (23.1) | 852 (23.4) |  | 44822 (52.1) |  |
| **Family history of lung cancer** |  | 0.288 |  | 0.288 |  | 1.397 |
| No | 2098 (39.6) | 860 (49) | 1238 (34.9) |  | 72753 (93.6) |  |
| Yes | 3200 (60.4) | 895 (51) | 2305 (65.1) |  | 4959 (6.4) |  |
| **Baseline comorbidity** |  |  |  |  |  |  |
| **Chronic respiratory diseases** |  |  |  | -0.046 |  | 3.741 |
| No | 333 (6.1) | 99 (5.4) | 234 (6.4) |  | 81038 (94.3) |  |
| Yes | 5150 (93.9) | 1748 (94.6) | 3402 (93.6) |  | 4934 (5.7) |  |
| **Digestive diseases** |  |  |  | 0.062 |  | 0.883 |
| No | 2158 (39.4) | 764 (41.4) | 1394 (38.3) |  | 67957 (79) |  |
| Yes | 3325 (60.6) | 1083 (58.6) | 2242 (61.7) |  | 18015 (21) |  |
| **Hepatobiliary diseases** |  |  |  | 0.209 |  | 0.848 |
| No | 2233 (40.7) | 878 (47.5) | 1355 (37.3) |  | 67926 (79) |  |
| Yes | 3250 (59.3) | 969 (52.5) | 2281 (62.7) |  | 18046 (21) |  |
| **Hypertension** |  |  |  | -0.008 |  | 0.102 |
| No | 3254 (68.4) | 1092 (68.1) | 2162 (68.5) |  | 57017 (73) |  |
| Yes | 1506 (31.6) | 511 (31.9) | 995 (31.5) |  | 21099 (27) |  |
| **Hyperlipidemia** |  |  |  | 0.073 |  | 0.43 |
| No | 3174 (66.7) | 1106 (69) | 2068 (65.6) |  | 66207 (84.8) |  |
| Yes | 1583 (33.3) | 497 (31) | 1086 (34.4) |  | 11904 (15.2) |  |
| **Diabetes** |  |  |  | -0.038 |  | 0.069 |
| No | 4301 (90.4) | 1437 (89.6) | 2864 (90.8) |  | 72116 (92.3) |  |
| Yes | 457 (9.6) | 166 (10.4) | 291 (9.2) |  | 5998 (7.7) |  |
| ^a^ Data were presented as n (%) unless otherwise specified.  ^b^ Non-smokers: 152 participants without information on body mass index, 129 participants without information on passive smoking, 8445 participants without information on family history of lung cancer, 8579 participants without information on hypertension, 8587 participants without information on hyperlipidemia, and 8583 participants without information on diabetes.  ^c^ No males were included as high-risk.  ^d^ Standardized differences were calculated as the difference in means or proportions divided by a pooled estimate of the standard deviation, which is not sensitive to sample size and is useful in identifying meaningful differences. Standardized differences greater than 0.1 were considered meaningful. | | | | | | |

## 
