# Supplementary material for: Disparity in Lung Cancer Screening Among Smokers and Nonsmokers in China: Prospective Cohort Study
Source: JMIR Public Health Surveill. 2023 Mar 14;9:e43586. doi: 10.2196/43586 (PMC10131892; doi:10.2196/43586)
Supplement: Multimedia Appendix 2 [file publichealth_v9i1e43586_app2.docx]

## Table S2. Baseline characteristics of the study population of smokers ^a,b^

| **Characteristics** | **High-risk** | | | | **Low-risk (N=11 261)** | **Standardized difference ^c^** |
| --- | --- | --- | --- | --- | --- | --- |
|  | **Total** **(N =18 818)** | **Non-screened (N=10 933)** | **Screened**  **(N=7885)** | **Standardized difference ^c^** |  |  |
| **Demographic characteristics** | |  |  |  |  |  |
| **Age (years)** |  |  |  |  |  |  |
| 40-54 | 7217 (38.4) | 4438 (40.6) | 2779 (35.2) | 0.11 | 4565 (40.5) | 0.045 |
| 55-74 | 11601 (61.6) | 6495 (59.4) | 5106 (64.8) |  | 6696 (59.5) |  |
| **Sex** |  |  |  | -0.109 |  | -0.035 |
| Male | 18178 (96.6) | 10654 (97.4) | 7524 (95.4) |  | 10946 (97.2) |  |
| Female | 640 (3.4) | 279 (2.6) | 361 (4.6) |  | 315 (2.8) |  |
| **Education** |  |  |  | 0.018 |  | 0.203 |
| Low | 5304 (28.2) | 3057 (28) | 2247 (28.5) |  | 2462 (21.9) |  |
| Medium | 11540 (61.3) | 6707 (61.3) | 4833 (61.3) |  | 6972 (61.9) |  |
| High | 1974 (10.5) | 1169 (10.7) | 805 (10.2) |  | 1827 (16.2) |  |
| **Body mass index** |  |  |  | 0.068 |  | 0.052 |
| <18·5 | 445 (2.4) | 271 (2.5) | 174 (2.2) |  | 204 (1.8) |  |
| 18·5-24 | 10066 (53.6) | 5977 (54.8) | 4089 (51.9) |  | 5885 (52.3) |  |
| 24-28 | 6882 (36.6) | 3904 (35.8) | 2978 (37.8) |  | 4330 (38.5) |  |
| ≥28 | 1393 (7.4) | 759 (7) | 634 (8.1) |  | 826 (7.3) |  |
| **Lifestyle factors** |  |  |  |  |  |  |
| **Occupational exposure to hazardous substances** | | |  | 0.261 |  | 0.185 |
| No | 14712 (78.2) | 9044 (82.7) | 5668 (71.9) |  | 9605 (85.3) |  |
| Yes | 4106 (21.8) | 1889 (17.3) | 2217 (28.1) |  | 1656 (14.7) |  |
| **Passive smoking** |  |  |  | 0.121 |  | 0.323 |
| No | 5735 (30.7) | 3595 (33) | 2140 (27.5) |  | 5199 (46.2) |  |
| Yes | 12935 (69.3) | 7286 (67) | 5649 (72.5) |  | 6050 (53.8) |  |
| **Frequent exercise** |  |  |  | -0.012 |  | -0.541 |
| No | 13486 (71.7) | 7810 (71.4) | 5676 (72) |  | 5178 (46) |  |
| Yes | 5332 (28.3) | 3123 (28.6) | 2209 (28) |  | 6083 (54) |  |
| **Family history of lung cancer** |  |  |  | 0.362 |  | 0.473 |
| No | 13583 (78.3) | 8370 (84.7) | 5213 (69.8) |  | 9936 (94.2) |  |
| Yes | 3764 (21.7) | 1509 (15.3) | 2255 (30.2) |  | 616 (5.8) |  |
| **Baseline comorbidity** |  |  |  |  |  |  |
| **Chronic respiratory diseases** | |  |  | 0.367 |  | 0.546 |
| No | 13440 (71.4) | 8565 (78.3) | 4875 (61.8) |  | 13440 (91.8) |  |
| Yes | 5378 (28.6) | 2368 (21.7) | 3010 (38.2) |  | 921 (8.2) |  |
| **Digestive diseases** |  |  |  | 0.281 |  | 0.223 |
| No | 12246 (65.1) | 7727 (70.7) | 4519 (57.3) |  | 8471 (75.2) |  |
| Yes | 6572 (34.9) | 3206 (29.3) | 3366 (42.7) |  | 2790 (24.8) |  |
| **Hepatobiliary diseases** |  |  |  | 0.343 |  | 0.175 |
| No | 12186 (64.8) | 7825 (71.6) | 4361 (55.3) |  | 8204 (72.9) |  |
| Yes | 6632 (35.2) | 3108 (28.4) | 3524 (44.7) |  | 3507 (27.1) |  |
| **Hypertension** |  |  |  | 0.089 |  | 0.016 |
| No | 11319 (66.4) | 6689 (68.2) | 4630 (63.9) |  | 7182 (67.1) |  |
| Yes | 5736 (33.6) | 3124 (31.8) | 2612 (36.1) |  | 3517 (32.9) |  |
| **Hyperlipidemia** |  |  |  | 0.203 |  | 0.076 |
| No | 13458 (78.9) | 8091 (82.4) | 5367 (74.1) |  | 8764 (81.9) |  |
| Yes | 3596 (21.1) | 1723 (17.6) | 1873 (25.9) |  | 1935 (18.1) |  |
| **Diabetes** |  |  |  | 0.084 |  | 0.016 |
| No | 15225 (89.3) | 8869 (90.4) | 6356 (87.8) |  | 9604 (89.8) |  |
| Yes | 1829 (10.7) | 944 (9.6) | 885 (12.2) |  | 1095 (10.2) |  |
| ^a^ Data were presented as n (%) unless otherwise specified.  ^b^ Smokers: 48 participants without information on body mass index, 160 participants without information on passive smoking, 2180 participants without information on family history of lung cancer, 2325 participants without information on hypertension, 2326 participants without information on hyperlipidemia, and 2326 participants without information on diabetes.  ^c^ Standardized differences greater than 0.1 were considered meaningful. | | | | | | |
