# Supplementary material for: Disparity in Lung Cancer Screening Among Smokers and Nonsmokers in China: Prospective Cohort Study
Source: JMIR Public Health Surveill. 2023 Mar 14;9:e43586. doi: 10.2196/43586 (PMC10131892; doi:10.2196/43586)
Supplement: Multimedia Appendix 3 [file publichealth_v9i1e43586_app3.docx]

**Table S3. Effect of risk evaluation and LDCT screening on lung cancer incidence, mortality and all-cause mortality among smokers**

|  | | Lung cancer incidence | |  | Lung cancer mortality | |  | All-cause mortality | |
| --- | --- | --- | --- | --- | --- | --- | --- | --- | --- |
|  |  | HR (95% CI) | *P* value |  | HR (95% CI) | *P* value |  | HR (95% CI) | *P* value |
| **Risk evaluation** | |  |  |  |  |  |  |  |  |
|  | Low-risk | 1.00 (reference) |  |  | 1.00 (reference) |  |  | 1.00 (reference) |  |
|  | High-risk | 2.28 (1.79-2.90) | <.001 |  | 2.79 (1.52-5.12) | <0.001 |  | 1.41 (1.04-1.89) | .025 |
|  | Adjusted HR^a^ | 2.17 (1.70-2.77) | <.001 |  | 2.77 (1.51-5.08) | 0.001 |  | 1.40 (1.04-1.88) | .027 |
|  | Adjusted HR^b^ | 2.20 (1.72-2.82) | <.001 |  | 2.93 (1.60-5.37) | <0.001 |  | 1.42 (1.06-1.91) | .02 |
| **LDCT screening** | |  |  |  |  |  |  |  |  |
|  | Non-screening | 1.00 (reference) |  |  | 1.00 (reference) |  |  | 1.00 (reference) |  |
|  | Screening | 1.41 (1.12-1.78) | .003 |  | 0.51 (0.28-0.94) | 0.032 |  | 0.51 (0.35-0.75) | <.001 |
|  | Adjusted HR^a^ | 1.40 (1.11-1.78) | .005 |  | 0.48 (0.26-0.89) | 0.020 |  | 0.48 (0.33-0.70) | <.001 |
|  | Adjusted HR^b^ | 1.39 (1.09-1.76) | .007 |  | 0.52 (0.28-0.96) | 0.037 |  | 0.47 (0.32-0.69) | <.001 |
| ^a^ Estimates are adjusted for demographic characteristics (sex, age, body mass index, education), lifestyle factors (occupational exposure to hazardous substances, passive smoking, frequent exercise) and family history of lung cancer. ^b^ Estimates are further adjusted for baseline comorbidity (chronic respiratory diseases, digestive diseases, hepatobiliary diseases, hypertension, hyperlipidemia, diabetes).  HR, hazard ratio; CI: confidential interval. | | | | | | | | | |
